# Predicting patient-specific quality assurance outcomes in helical tomotherapy using plan complexity and 3D dose-distribution radiomics

**DOI:** 10.3389/fonc.2026.1883024

**Published:** 2026-07-20

**Authors:** Xiaoli Jin, Xiaoguang Xiao, Rui Guo, Tao Chen, Cairong Hu

**Affiliations:** 1 Department of Oncology, Shanxi Bethune Hospital, Shanxi Academy of Medical Sciences, Tongji Shanxi Hospital, Third Hospital of Shanxi Medical University, Taiyuan, China; 2 Department of Oncology, Tongji Hospital of Tongji Medical College, Huazhong University of Science and Technology, Wuhan, China; 3 Department of Radiation Oncology, Clinical Oncology School of Fujian Medical University, Fujian Cancer Hospital, Fuzhou, China; 4 Shanghai Institute of Applied Physics, Chinese Academy of Sciences, Shanghai, China; 5 University of Chinese Academy of Sciences, Beijing, China

**Keywords:** gamma pass rate, helical tomotherapy, machine learning, patient-specific quality assurance, plan complexity, radiomic features

## Abstract

Pretreatment patient-specific quality assurance (PSQA) for helical tomotherapy (HT) is time-consuming and resource-intensive. Machine-learning-based screening tools may help prioritize measurement-based QA resources. This study investigated whether combining plan complexity descriptors with 3D dose-distribution radiomic features could improve the prediction of HT plan deliverability. A total of 498 clinical HT plans from two institutions were retrospectively analyzed, including 286 plans from Institution 1 and 212 from Institution 2. For each plan, 72 plan complexity features and 851 dose-distribution radiomic features were extracted. Support vector machine classifiers were developed separately for each institution using three feature sets: plan complexity features alone, dose-distribution radiomic features alone, and their combination. Model performance was evaluated on independent test sets using receiver operating characteristic analysis, with ground−truth PSQA outcomes defined according to preset gamma passing−rate thresholds. Across both institutions and both gamma criteria, the hybrid model achieved the best discrimination. Its AUCs were 0.774 and 0.938 for γ 3%/2 mm in Institutions 1 and 2, respectively, and 0.820 and 0.825 for γ 2%/2 mm. The most informative predictors included variables from both feature domains, and the hybrid model achieved higher AUCs than models using either domain alone. Additional analyses, including an exact permutation test and a bidirectional cross-institution evaluation, showed that the high AUCs were unlikely to result from chance but highlighted limited transferability across centers. These findings suggest that integrating plan complexity and 3D dose-distribution radiomic features may support risk-adapted pretreatment PSQA screening for HT. However, external multi-institution validation and prospective workflow assessment are required before considering clinical implementation.

## 1 Introduction

Helical tomotherapy (HT) is a highly modulated radiotherapy technique that can produce conformal dose distributions while sparing surrounding organs at risk (1). However, the high degree of modulation may also introduce delivery uncertainties. Rigorous pretreatment dose verification is therefore needed to confirm calculation accuracy and detect potential errors (2). As a result, patient-specific quality assurance (PSQA) remains an essential part of treatment plan validation (3, 4).

Conventional PSQA relies on phantom measurements and gamma analysis (5, 6). This approach is widely used in clinical practice, but it is time-consuming and resource-intensive. It is also not well suited to adaptive radiotherapy workflows. For these reasons, predictive models have increasingly been explored as decision-support tools to complement measurement-based PSQA (7–17). Most machine-learning studies in this area have focused on intensity-modulated radiotherapy (IMRT) and volumetric modulated arc therapy (VMAT) (7–16). In contrast, similar work for HT is still limited (17).

In recent years, dedicated complexity metrics for HT have been proposed and refined. Santos et al. (18) introduced HT-specific complexity metrics, and later studies by Cavinato and colleagues further standardized their extraction through the TCoMX framework (19, 20). Plan complexity is considered one of the factors most closely related to HT delivery performance (18, 19, 21). Based on this concept, Cavinato et al. (17) reported encouraging results for HT virtual PSQA using delivery parameters, complexity metrics, and sinogram radiomics features. In HT, a sinogram is a two-dimensional representation of MLC opening and closing during gantry rotation (1). Therefore, the features used in that study mainly reflected delivery- and MLC-related properties. Although encouraging, the reported performance also suggested room for further improvement. Studies in VMAT have suggested that combining complexity-related variables with quantitative dose-based descriptors can improve prediction performance (9, 10, 14). Three-dimensional dose-distribution features can capture the shape, intensity pattern, and spatial heterogeneity of the planned dose delivered to targets and organs at risk. However, it remains unclear whether adding 3D dose-distribution features could improve prediction performance in HT.

To address this gap, we developed machine-learning-based binary classification models for HT PSQA by integrating HT-specific plan complexity features with 3D dose-distribution radiomic features. Unlike previous HT virtual PSQA studies that mainly focused on delivery or sinogram-derived information, this study evaluated whether planned 3D dose-distribution characteristics could provide additional predictive value.

## 2 Materials and methods

### 2.1 Dataset

**Figure 1:**
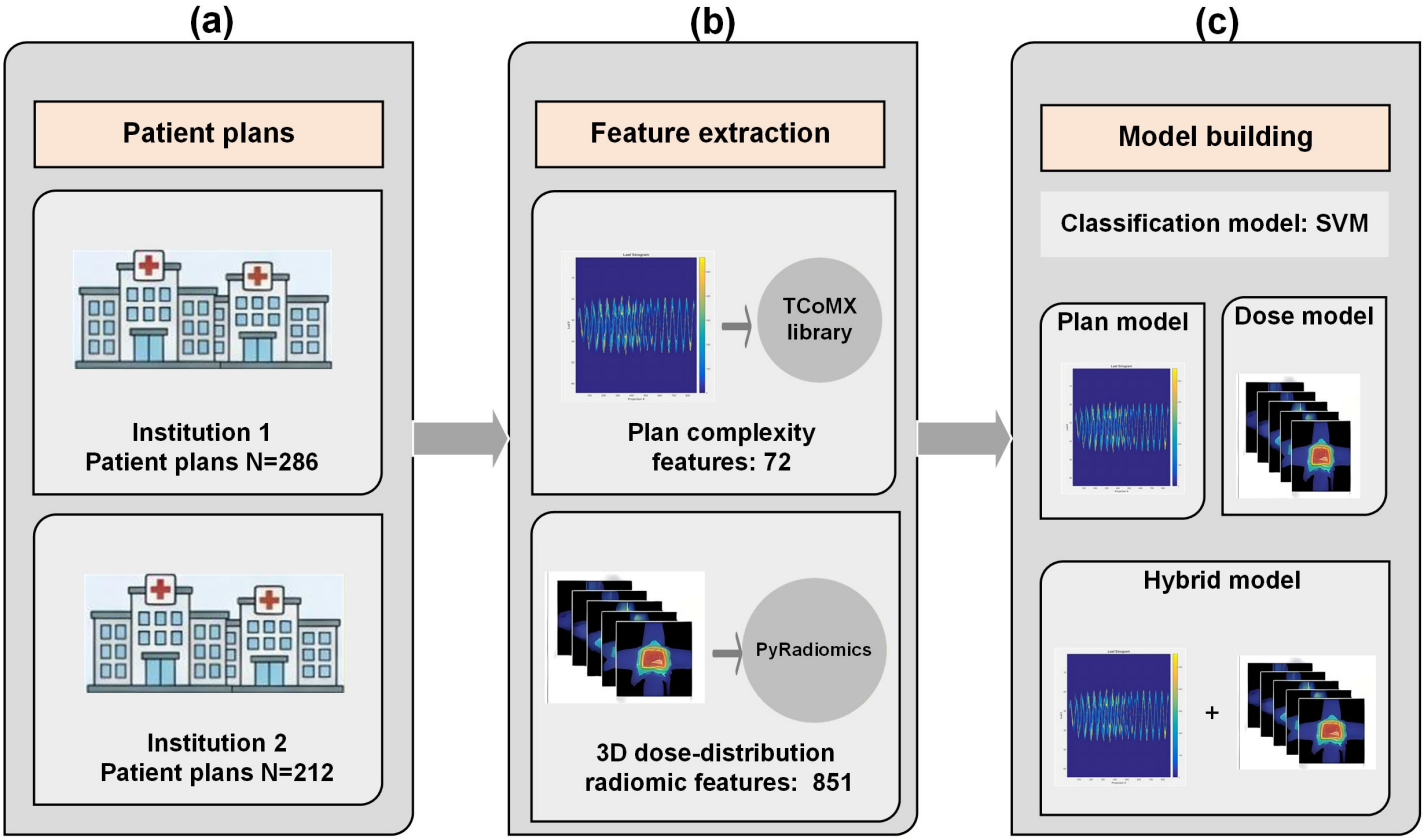
Overview of the study design, feature extraction, and model framework. **(a)** Study cohort including 498 helical tomotherapy plans from two institutions. **(b)** Extraction of 72 plan complexity features using the TCoMX library and 851 3D dose-distribution radiomic features using PyRadiomics. **(c)** Construction of three feature-based models: the plan model, dose model, and hybrid model.

This retrospective study included 498 clinical HT treatment plans collected from two institutions between September 2019 and March 2023 (**Figure 1a**). Institution 1 contributed 286 plans, and Institution 2 contributed 212 plans. Plans from Institution 1 were generated on TomoH systems, whereas plans from Institution 2 were generated on RadixAct systems. Treatment planning was performed using Accuray Planning Station version 5.1.8.23 for TomoH and Accuray Precision version 2.0.1.1 for RadixAct. Both systems used the GPU-based VoLO optimizer and the collapsed cone convolution superposition algorithm for dose calculation.

The cohort included multiple treatment sites, such as brain, thorax, liver, and prostate. The prescribed dose per fraction ranged from 1.8 to 6 Gy. All plans had undergone routine institutional PSQA measurements before treatment according to local clinical practice.

### 2.2 PSQA measurements

All plans underwent measurement-based PSQA using the ArcCHECK detector array (Sun Nuclear Corporation, Melbourne, FL, USA) with a PMMA CavityPlug. For phantom-based verification, megavoltage CT images with a slice thickness of 2 mm were acquired for phantom modeling. Each treatment plan was then transferred to the modeled phantom in the delivery quality assurance workstation for dose recalculation. The calculated dose distribution was exported to the ArcCHECK software via DICOM. After delivery to the phantom, the measured and calculated absolute dose distributions were compared.

Global gamma analysis was performed using absolute dose comparison with a 10% low-dose threshold. Two gamma criteria were evaluated: 3%/2 mm and 2%/2 mm. The same ArcCHECK-based workflow and gamma evaluation settings were used at both institutions.

### 2.3 Feature extraction

A total of 923 features were extracted from each plan, including 72 plan complexity features and 851 3D dose-distribution radiomic features (**Figure 1b**). Plan complexity features were extracted from CT images and DICOM-RT plan files using the TCoMX library (19). These features described several aspects of HT delivery, including field geometry, beam modulation, and leaf open time (LOT) behavior.

3D dose-distribution radiomic features were extracted from CT images and DICOM-RT dose files. Before feature extraction, dose distributions were converted to prescription-normalized relative dose and processed with a consistent PyRadiomics configuration for all cases (22). The radiomic extraction mask was defined as a single unified 3D ROI including all voxels receiving at least 10% of the prescription dose. Gray-level discretization was performed using a fixed bin width of 0.1 (in relative dose units), and both wavelet-filtered and original features were extracted. No case-specific adjustment of radiomic extraction parameters was performed.

Radiomic features characterized the morphology, intensity statistics, and spatial heterogeneity of the planned dose distribution. Seven feature categories were computed: shape, first-order statistics, gray level dependence matrix (GLDM), gray level co-occurrence matrix (GLCM), gray level run length matrix (GLRLM), gray level size zone matrix (GLSZM), and neighboring gray-tone difference matrix (NGTDM). A complete feature list is provided in **Supplementary Table 1**.

Three feature sets were used for model development (**Figure 1c**): plan complexity features alone, referred to as the plan model (PM); 3D dose-distribution radiomic features alone, referred to as the dose model (DM); and the combination of both feature domains, referred to as the hybrid model (HM).

### 2.4 Data preprocessing and label definition

Data preprocessing was performed separately for the two institutional datasets, and all analyses were conducted independently within each institution. No plans had missing feature values. For each institutional dataset, the plans were randomly divided into a training set (80%) and an independent test set (20%) using stratified sampling based on the PSQA class labels to preserve the proportion of threshold-passing and threshold-failing plans. A fixed random seed of 42 was used. To avoid data leakage, all plans from the same patient were assigned to the same subset. Stratification by treatment site was not applied because, even for sites with sufficient case numbers, the number of threshold-failing plans was too small, leading to unstable class distributions and unreliable model performance.

Feature standardization, feature cleaning, recursive feature elimination, and hyperparameter optimization were performed using only the training data. The fitted preprocessing parameters were then applied to the test set, which was used exclusively for final model evaluation.

The prediction task was formulated as a binary classification based on measured gamma pass rate (GPR) thresholds following AAPM Task Group 218 (4). A plan was labeled as “passing the PSQA threshold” if its measured GPR met or exceeded the tolerance limit (95% for γ 3%/2 mm and 90% for γ 2%/2 mm); otherwise, it was labeled as “failing the PSQA threshold”.

### 2.5 Model development

**Figure 2:**
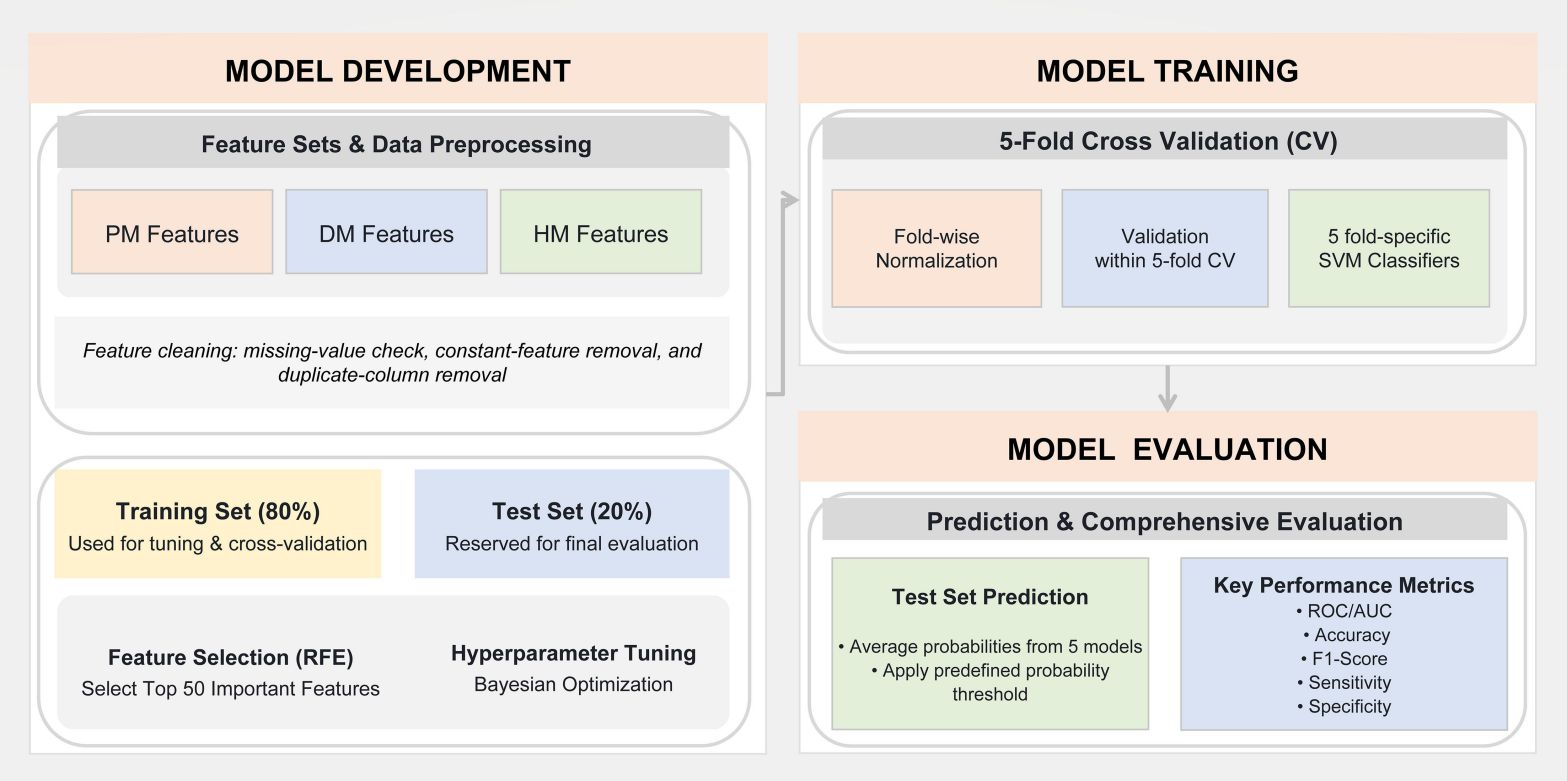
Workflow of SVM-based model development, training, and evaluation for HT PSQA classification. Model development included definition of three feature sets (plan model, dose model, and hybrid model), data preprocessing, and an 80/20 stratified split into training and independent test sets. Within the training set, feature selection using recursive feature elimination (RFE) and Bayesian optimization of SVM hyperparameters were performed. Model training was carried out using 5-fold cross-validation with fold-wise normalization, yielding five fold-specific SVM classifiers. For model evaluation, predicted probabilities from the five classifiers were averaged for each test plan, and discrimination performance was assessed using ROC/AUC, accuracy, F1-score, sensitivity, and specificity.

Support vector machine (SVM) classifiers were developed to predict whether HT plans would pass the predefined PSQA thresholds. Modeling was performed separately for each institution, gamma criterion, and feature set. The overall workflow is shown in **Figure 2**.

Feature selection was conducted within the training subset using recursive feature elimination (RFE) with a linear−kernel SVM as the base estimator. To determine the appropriate number of retained features, a sensitivity analysis was performed by varying the number of selected features from 30 to 70 and evaluating performance on the training cross−validation folds. As shown in **Supplementary Figure 1**, the validation error reached a minimum at 50 features; therefore, the top 50 ranked features were used for model development. A regression−based surrogate loss (MAE) was used because it yielded more stable feature−ranking behavior in preliminary testing than classification−based losses under the highly imbalanced labels.

SVM hyperparameters were optimized on the training subset using Bayesian optimization based on the tree-structured Parzen estimator algorithm. The search space included the penalty parameter C, kernel type (linear, radial basis function, or polynomial), and the kernel coefficient γ for non-linear kernels. Optimization was performed using five-fold cross-validation with AUC as the objective function and a maximum of 50 iterations.

After hyperparameter tuning, a second round of five−fold cross−validation was performed on the training subset to train five fold−specific SVM classifiers. Within each fold, a z−score standardization was fitted on the fold’s training portion and then applied to its validation portion as well as to the independent test subset. The test subset was never used for feature selection, hyperparameter tuning, or fitting the scaler. Each fold−specific classifier produced predicted probabilities for all samples in the test subset, and the final probability for each test sample was obtained by averaging the five fold−specific predictions. Probability estimates were derived using Platt scaling as implemented in the SVM classifier.

Because the class distribution was markedly imbalanced, preliminary experiments were also performed using imbalance-handling strategies, including class weighting and undersampling. However, these approaches did not consistently improve performance on the independent test sets across institutions and gamma criteria. Therefore, the final analyses were based on the original class distributions.

To further assess model robustness and cross−institution generalizability, two additional validation analyses were performed: permutation testing and cross−institution evaluation.

### 2.6 Performance evaluation

Model performance was evaluated on the independent test subset using receiver operating characteristic (ROC) analysis and the area under the ROC curve (AUC). Sensitivity, specificity, accuracy, and F1-score were calculated using a probability cutoff of 0.5 for the classifier output. The GPR action limits were used to generate binary reference labels. Because of the skewed class distribution, AUC was considered the primary summary measure of discrimination performance. AUCs were reported with 95% confidence intervals estimated by 1000 bootstrap resamples.

## 3 Results

### 3.1 Dataset statistics

The distributions of measured gamma pass rates are summarized in **Table 1**. In both institutions, GPR values were strongly skewed toward high values, particularly under the γ 3%/2 mm criterion. For γ 3%/2 mm, most plans had GPRs between 95% and 100%, accounting for 89.9% of plans in Institution 1 and 94.8% in Institution 2. Under the stricter γ 2%/2 mm criterion, the distributions were broader, but the majority of plans still remained above the selected action limit.

**Table 1:** Distribution of measured gamma pass rates in the two institutions under the γ 3%/2 mm and γ 2%/2 mm criteria.

Measured GPR	γ 3%/2 mm		γ 2%/2 mm	
	Institution 1	Institution 2	Institution 1	Institution 2
Mean ± SD (%)	97.9 ± 2.4	98.7 ± 1.8	94.3 ± 4.7	96.0 ± 3.5
95-100 (n (%))	257 (89.9%)	201 (94.8%)	155 (54.2%)	149 (70.3%)
90-94 (n (%))	22 (7.7%)	10 (4.7%)	87 (30.4%)	48 (22.6%)
85-89 (n (%))	7 (2.5%)	1 (0.5%)	29 (10.1%)	12 (5.7%)
80-84 (n (%))	0	0	9 (3.2%)	3 (1.4%)
< 80 (n (%))	0	0	6 (2.1%)	0

The table summarizes gamma pass rate (GPR) distributions for all 498 plans included in the study. Values reflect the measured ArcCHECK-based PSQA results using the 95% action limit for γ 3%/2 mm and the 90% action limit for γ 2%/2 mm. GPR, gamma pass rate.

### 3.2 Importance of the predictors

In the hybrid model, both feature domains were retained among the top 50 predictors across institutions and gamma criteria (**Table 2**). Plan complexity metrics contributed 6–13 of the selected features, while 37–44 were 3D dose-distribution radiomic features. Within the radiomic domain, wavelet-filtered descriptors were more frequently selected than original features.

**Table 2:** Distribution of feature domains among the 50 features selected for the hybrid model.

Institution	Gamma criteria	Plan complexity (n)	Dose radiomics (n)	Original (n)	Wavelet (n)
Institution 1	γ 3%/2mm	12	38	5	33
Institution 1	γ 2%/2mm	13	37	8	29
Institution 2	γ 3%/2mm	6	44	8	36
Institution 2	γ 2%/2mm	12	38	6	32

Counts represent the number of plan-complexity and dose-radiomic features retained by recursive feature elimination for each institution and gamma criterion. Dose-radiomic features include both original and wavelet-filtered descriptors.

The five highest ranked predictors for each setting are listed in **Table 3**. Most were radiomic texture features, including wavelet-based GLCM, GLDM, GLSZM, and NGTDM descriptors. Such features capture spatial correlations and heterogeneity within the planned 3D dose distribution. Several plan complexity metrics, such as minFLOT, sdFLOT, and nCC, also appeared among the top predictors.

**Table 3:** Five highest−ranked predictors of the hybrid model for each institution and gamma criterion.

Gamma criteria	Institution 1		Institution 2	
	Feature	Feature type	Feature	Feature type
HM (γ 3%/2mm)	wavelet-LLH_glcm_Correlation	Dose radiomics	minFLOT	Plan complexity
	original_firstorder_Maximum	Dose radiomics	original_gldm_DependenceEntropy	Dose radiomics
	wavelet-HLH_glcm_Correlation	Dose radiomics	nCC	Plan complexity
	wavelet-HLH_ngtdm_Complexity	Dose radiomics	wavelet-LLL_gldm_DependenceEntropy	Dose radiomics
	wavelet-HHL_firstorder_Skewnes	Dose radiomics	wavelet-LLH_firstorder_Maximum	Dose radiomics
HM (γ 2%/2mm)	wavelet-HHH_ngtdm_Complexity	Dose radiomics	minFLOT	Plan complexity
	sdFLOT	Plan complexity	wavelet-HHL_firstorder_TotalEnergy	Dose radiomics
	wavelet-HHL_glcm_ClusterProminence	Dose radiomics	wavelet-HHL_firstorder_Energy	Dose radiomics
	original_shape_Maximum2DDiameterColumn	Dose radiomics	wavelet-HHL_glszm_SizeZoneNonUniformity	Dose radiomics
	original_glrlm_LongRunHighGrayLevelEmphasis	Dose radiomics	wavelet-HHH_glcm_MCC	Dose radiomics

Feature importance rankings were derived from the hybrid SVM models trained within each institution. Feature types correspond to plan−complexity metrics or 3D dose−distribution radiomic descriptors. HM, hybrid model.

### 3.3 Classification performance

**Figure 3:**
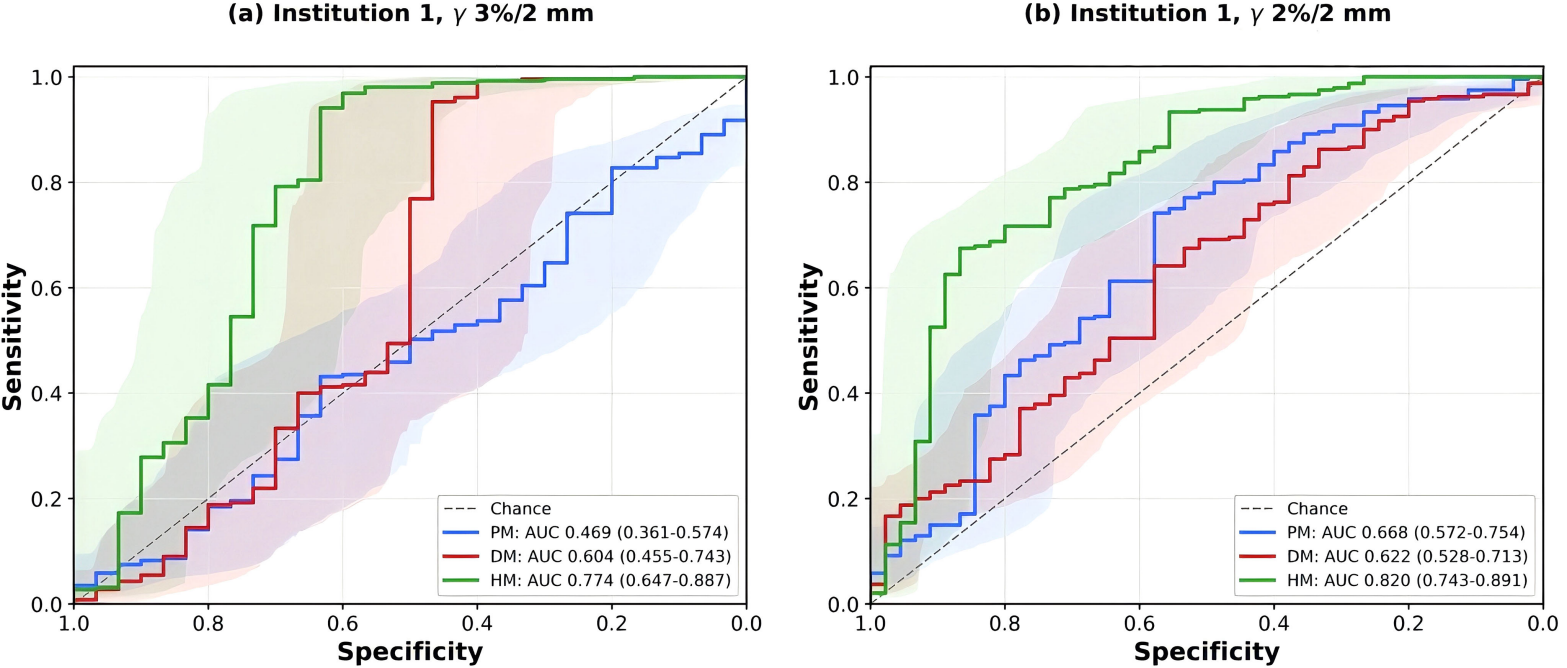
ROC curves of the three classification models in Institution 1. **(a)** γ 3%/2 mm with a 95% action limit. **(b)** γ 2%/2 mm with a 90% action limit. Solid lines show the mean ROC curves for each model, and the shaded areas indicate the corresponding 95% confidence intervals. The diagonal dashed line (“Chance”) represents the performance of a random classifier (AUC , 0.5).

**Figure 4:**
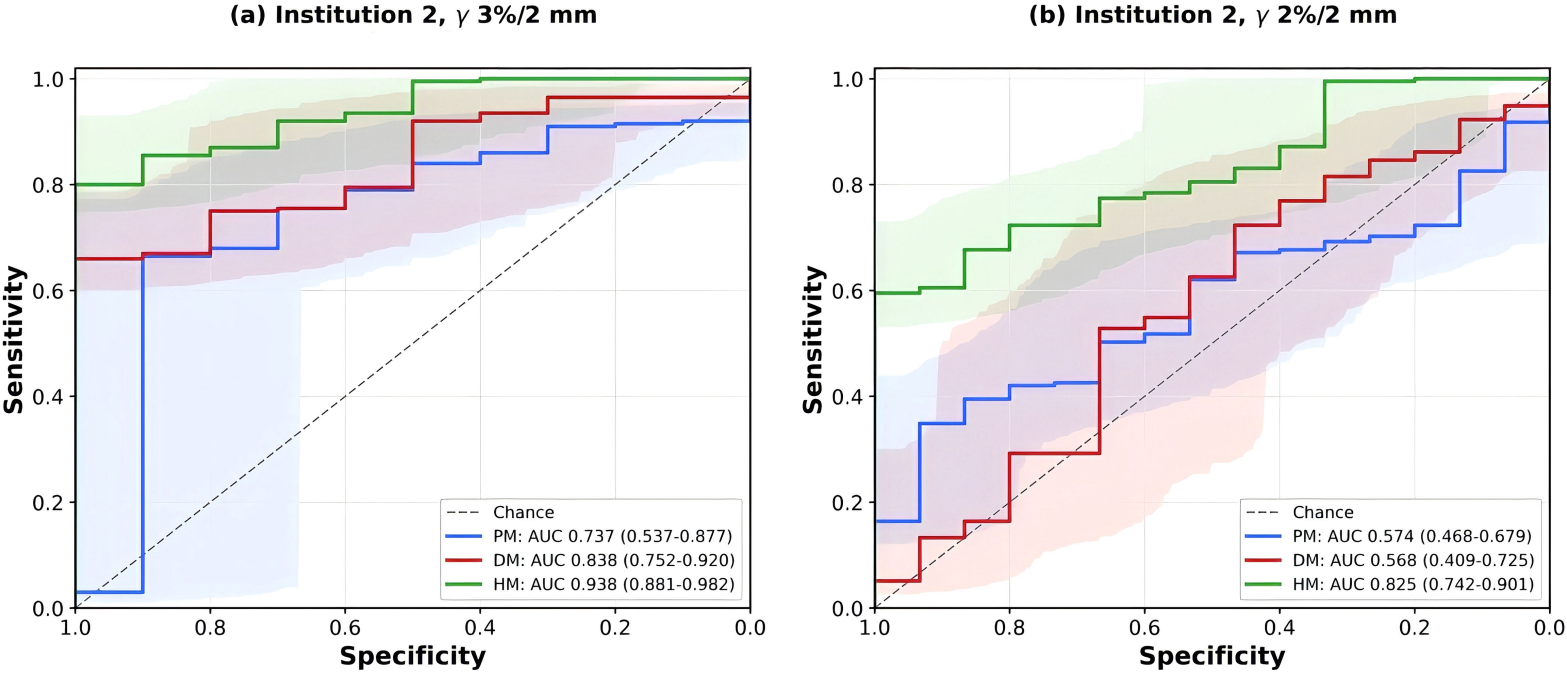
ROC curves of the three classification models in Institution 2. **(a)** γ 3%/2 mm with a 95% action limit. **(b)** γ2%/2 mm with a 90% action limit. Solid lines show the mean ROC curves for each model, and the shaded areas indicate the corresponding 95% confidence intervals. The diagonal dashed line (“Chance”) represents the performance of a random classifier (AUC , 0.5).

ROC curves for Institutions 1 and 2 are shown in **Figures 3** and **4**, and the detailed performance metrics are summarized in **Supplementary Tables 2**, **3**. Across both institutions and gamma criteria, the hybrid model achieved the highest AUCs among the three evaluated feature sets. For the γ 3%/2 mm criterion, AUCs were 0.774 and 0.938 in Institutions 1 and 2, respectively. For the γ 2%/2 mm criterion, the hybrid model reached AUCs of 0.820 and 0.825 in the two institutions.

The class imbalance in both datasets resulted in uniformly high sensitivities and comparatively low specificities across all models. Despite this, the hybrid model consistently exhibited the highest overall discrimination, with improvements in AUC relative to the plan−only and dose−only models. At 100% sensitivity under the γ 3%/2 mm criterion, the hybrid model identified approximately 33% and 50% of threshold−passing plans in Institutions 1 and 2, representing the proportion of passing plans that the model could confidently classify while preserving perfect sensitivity.

Supplementary analyses, including the permutation testing and cross−institution evaluation, are reported in **Supplementary Tables 4**, **5**.

## 4 Discussion

This study investigated machine-learning-based classification of HT PSQA outcomes using plan complexity features, 3D dose-distribution radiomic features, and their combination. The main finding was that the hybrid model consistently achieved the best discrimination performance across both institutions and under both γ 3%/2 mm and γ 2%/2 mm criteria. These results suggest that incorporating both complexity descriptors and dose−distribution features improves predictive performance compared with using either feature domain alone.

Plan complexity has long been linked to treatment deliverability, and recent studies have shown that quantitative image− and dose−derived features can provide additional predictive value (9–14, 16). In HT, complexity metrics capture machine− and delivery−related characteristics such as modulation behavior, field geometry, and LOT statistics (18, 20). In contrast, 3D dose−distribution radiomic features describe the morphology, intensity patterns, and spatial heterogeneity of the planned dose distribution (22). In this study, integrating these two feature domains yielded the highest discrimination performance across institutions and gamma criteria. The feature importance analysis provides insight into why the hybrid strategy performed best. Many of the top-ranked predictors were wavelet-based radiomic texture features, indicating that multi−scale and directional dose−texture information played a substantial role in differentiating plans that passed or failed PSQA thresholds. These features capture structured spatial variations in dose that may reflect underlying modulation complexity. At the same time, several plan complexity metrics were consistently selected, particularly those related to LOT distributions and sinogram-derived modulation behavior. These variables quantify aspects of delivery dynamics that are not represented in the dose distribution itself. For example, minFLOT reflects extremely short leaf opening intervals that can challenge mechanical timing and thus increase the risk of PSQA failure.

This pattern may help contextualize the present findings relative to previous work. Cavinato et al. (17) reported encouraging results for HT virtual PSQA using a hybrid model. Their model combined delivery parameters, complexity metrics, and sinogram radiomics features. Because a sinogram is a two-dimensional representation of MLC opening and closing during gantry rotation (1), that approach mainly captured delivery- and MLC-related information. By incorporating 3D dose−distribution radiomic features, the present study introduces a distinct and spatially informative feature domain that was not available in previous HT models. Although the datasets and evaluation endpoints differed, the higher AUC values observed in the present work (0.82 and 0.938 vs. 0.69 and 0.80 in Cavinato et al.) suggest that adding 3D dose-distribution descriptors may further enhance predictive performance when combined with delivery-related information. This result aligns with findings in VMAT showing that combining modulation metrics with dose-texture descriptors can improve predictive performance (9, 10, 14).

A characteristic pattern of the present results was high sensitivity combined with limited specificity. This was likely related to the marked class imbalance in both institutional datasets, where most plans had high gamma pass rates. Similar findings have been reported in other PSQA prediction studies (23, 24). Despite this challenge, the hybrid model consistently achieved the highest AUC and the best specificity among the tested models. This suggests that combining the two feature domains can improve discrimination even when class distributions are highly skewed. At a fixed sensitivity of 100%, the specificities of the hybrid model were 0.33 and 0.50 for Institutions 1 and 2, respectively. For comparison, Cavinato et al. (17) reported corresponding values of 0.16 and 0.63 for TPS1 and TPS2. These results highlight the practical difficulty of maintaining perfect sensitivity while preserving specificity when clinically measured gamma pass rates are used as the reference standard.

Given the particularly high AUC in Institution 2 under the γ 3%/2 mm criterion, we conducted an exact permutation test to evaluate whether this result could arise from random label configurations. The observed AUC of 0.9375 was significantly above the permutation distribution (p , 0.0139), indicating that the model’s signal is unlikely to be purely due to chance. However, the small number of failing plans means that the estimate remains sensitive to sample composition, underscoring the need for further validation on larger datasets.

It is important to note that any virtual PSQA approach fundamentally relies on a rigorous and stable machine QA program. The model evaluates plan- and dose-related characteristics only and cannot detect hardware issues such as output drift, MLC malfunction, or mechanical instability. Therefore, a plan predicted to meet PSQA thresholds cannot be considered clinically safe if machine QA performance deviates from tolerance. In addition, False-negative predictions may also occur for highly modulated or anatomically complex plans, highlighting the need to retain measurement−based QA for cases with greater delivery complexity or higher clinical priority.

The model is also inherently device specific. As highlighted by Kry et al (25)., PSQA results can vary substantially across detector systems, and gamma analysis metrics have known limitations related to detector geometry, sampling resolution, and their imperfect correlation with true delivery errors. Consequently, the present model primarily reflects the behavior of the ArcCHECK-based workflow used in this study and should not be assumed to generalize to other detectors or measurement systems without dedicated validation.

Furthermore, the additional cross-institution evaluation revealed a marked decline in performance in this study, indicating limited transferability. Differences in planning systems, delivery platforms, commissioning procedures, and PSQA workflows likely contributed to this degradation. Taken together, these findings suggest that the current model should be regarded as a device- and institution-specific decision support tool, rather than a replacement for machine QA or a universally applicable PSQA solution.

This study has several limitations. First, the class imbalance in both institutional datasets was not fully addressed. More effective strategies, including imbalance-aware learning methods or augmentation of failing cases, should be explored in future work. Second, even with the inclusion of a cross−institution evaluation, the substantial degradation in performance across centers demonstrates limited model transferability. Thus, the present results constitute institution−specific validation rather than a formal external validation across institutions. Third, the results are tied to the specific planning systems, delivery platforms, and ArcCHECK-based measurement workflows used in the participating institutions. The potential impact of other detector systems or workflow variations was not explicitly evaluated. Finally, because threshold-failing plans were too few within individual disease sites to support stable site-specific training, the potential impact of anatomical heterogeneity on feature distributions and model behavior remains unknown. Future studies should incorporate larger multicenter datasets and include measurements from multiple detector types and delivery platforms. Such efforts will be essential for establishing the generalizability of virtual PSQA and clarifying its safe and practical role in routine HT workflows.

## 5 Conclusion

In this retrospective study, combining HT plan complexity features with 3D dose-distribution radiomic features improved the discrimination of threshold-defined PSQA outcomes compared with either feature domain alone. With external validation and prospective workflow assessment, this approach may support risk-adapted pretreatment PSQA screening for helical tomotherapy.

## Data Availability

The raw data supporting the conclusions of this article will be made available by the authors, without undue reservation.

## Supplementary material

Supplementary Figure 1 Sensitivity analysis of the number of features selected by recursive feature elimination. Validation error was evaluated for different numbers of retained features. The minimum validation error occurred at 50 features, which was selected as the final feature count for model development.

Supplementary Table 1 Summary of plan complexity and 3D dose−distribution radiomic features.The table lists the feature categories, feature counts, and brief descriptions for all variables extracted in this study. Abbreviations correspond to the feature groups defined in the Methods. Abbreviations: GLCM, gray level co-occurrence matrix, GLDM, gray level dependence matrix, GLRLM, gray level run length matrix, GLSZM, gray level size zone matrix, NGTDM, neighboring gray tone difference matrix, FW, Field Width, PT, Projection Time, GP, Gantry Period, TT, Treatment Time, TL, Target Length, CS, Couch Speed, CT, Couch Translation, Nproj, Number of projections, Nrot, Number of rotations, MF, Modulation Factor, TTDF, Treatment time over fraction dose, mLOT, LOT mean, sdLOT, LOT standard deviation, mdLOT, LOT median, moLOT, LOT mode, minLOT, LOT minimum, maxLOT, LOT maximum, CLNSn, Cumulative LOT Number Score, CLNSpt,n, Cumulative LOT Number Score at Projection Time, kLOT, LOT kurtosis, sLOT, LOT skewness, mFLOT, sdFLOT, FLOT standard deviation, moFLOT, FLOT mode, mdFLOT, FLOT median, minFLOT, FLOT minimum, maxFLOT, FLOT maximum, CFNSn, Cumulative FLOT Number Score, LnNS, Leaves with n Open Nearest Neighbors, CLS, Closed Leaf Score, CLSin, Closed Leaf Score within the treatment area, nCC, Number of Connected Components, lengthCC, Length of the Connected Components, TA, Treatment Area, fDISC, Fraction of Discontinuous Projections, CLSin,area, the number of closed leaves within the treatment area, CLSin,disc, it is computed by considering the discontinuous projections is then divided by the number of discontinuous projections, CLSin,area,disc, the combination of the CLSin,area with the CLSin,disc, PSTV, Plan Sinogram Time Variation, LOTV, Leaf Open Time Variability, MI, Modulation Index, nOC, Number of Openings and Closures, EPSTV∆p,∆l, Extended Plan Sinogram Time Variation, mSI, the mean sinogram intensity, mdSI, the median sinogram intensity, sdSI, the standard deviation sinogram intensity, MSA, Mean Sinogram Asymmetry.

Supplementary Table 2 Classification performance and test-set class distribution for Institution 1. Results are shown for the 95% action limit under the γ 3%/2 mm criterion and the 90% action limit under the γ 2%/2 mm criterion. Positive and negative counts refer to the number of threshold-passing and threshold-failing plans in the independent test subset. Abbreviations: AUC, area under the receiver operating characteristic curve; PM, plan model; DM, dose model; HM, hybrid model.

Supplementary Table 3 Classification performance and test-set class distribution for Institution 2. Results are shown for the 95% action limit under the γ 3%/2 mm criterion and the 90% action limit under the γ 2%/2 mm criterion. Positive and negative counts refer to the number of threshold-passing and threshold-failing plans in the independent test subset. Abbreviations: AUC, area under the receiver operating characteristic curve; PM, plan model; DM, dose model; HM, hybrid model.

Supplementary Table 4 Exact permutation test results for the hybrid model in Institution 2 under the γ 3%/2 mm criterion. The permutation test assessed whether the high AUC observed for the hybrid model could arise from random label assignments under the highly imbalanced class distribution. The number of threshold−passing and threshold−failing labels in the test set was held constant (40 and 2, respectively), and all C(42, 2) , 861 possible label configurations were enumerated. For each configuration, the AUC was recalculated while keeping the model−predicted probabilities fixed. The one−sided p−value was defined as the proportion of permutations with AUC values greater than or equal to the observed AUC.

Supplementary Table 5 Cross-institution validation results : AUCs for models trained on one institution and tested on the other. For each direction, models were trained using the full dataset from the training institution only. The trained models were then directly applied to the complete dataset of the testing institution, which did not participate in model development. The γ 3%/2 mm and γ 2%/2 mm criteria correspond to action limits of 95% and 90%, respectively.
