# Supplementary figures and images for: Predicting patient-specific quality assurance outcomes in helical tomotherapy using plan complexity and 3D dose-distribution radiomics
Source: Front Oncol. 2026 Jul 20;16:1883024. doi: 10.3389/fonc.2026.1883024 (PMC13429479; doi:10.3389/fonc.2026.1883024)

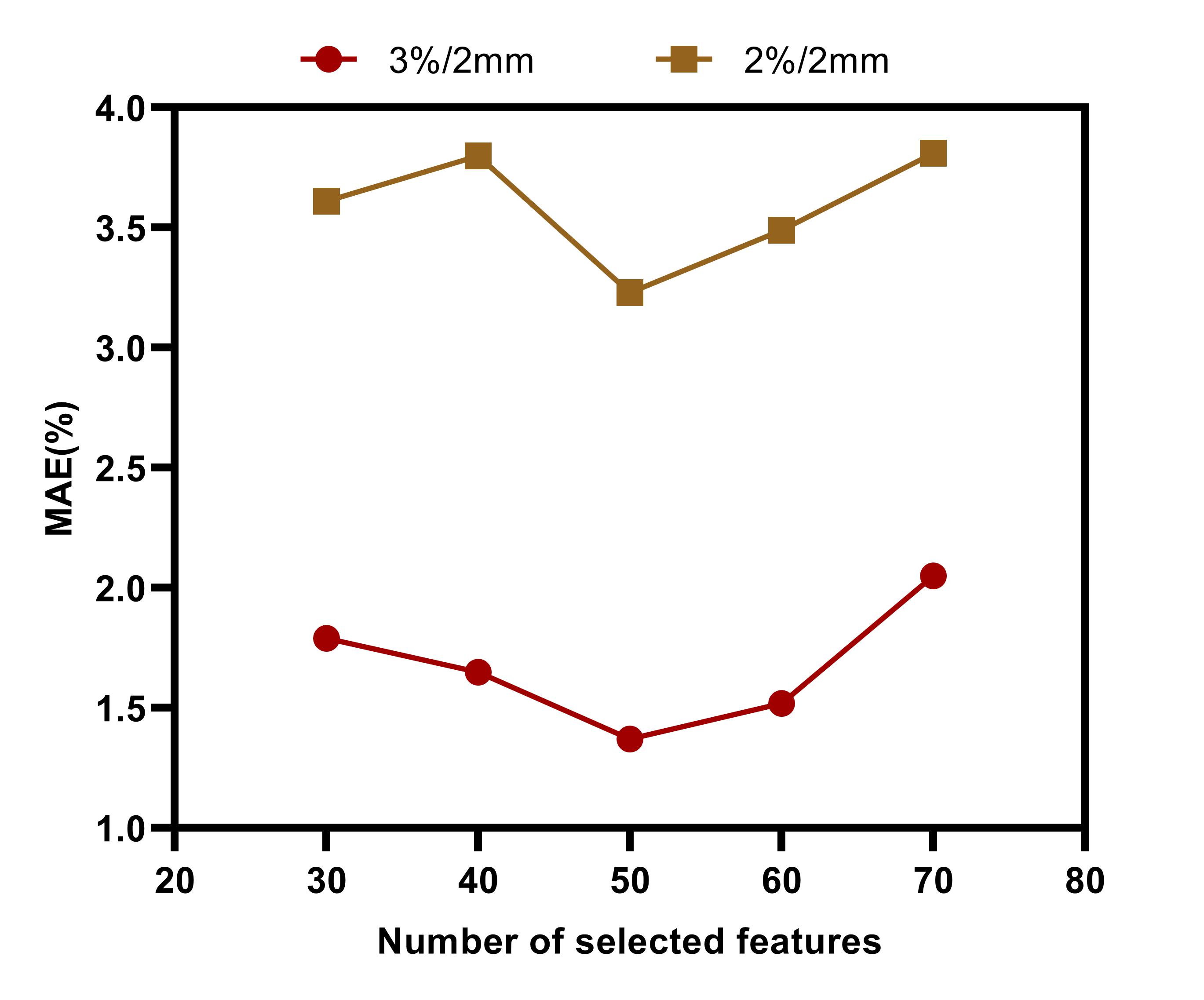

Supplement: Supplementary Figure 1 — Sensitivity analysis of the number of features selected by recursive feature elimination. Validation error was evaluated for different numbers of retained features. The minimum validation error occurred at 50 features, which was selected as the final feature count for model development. [file Supplementaryfile1.jpg]
