# Supplementary material for: Predicting patient-specific quality assurance outcomes in helical tomotherapy using plan complexity and 3D dose-distribution radiomics
Source: Front Oncol. 2026 Jul 20;16:1883024. doi: 10.3389/fonc.2026.1883024 (PMC13429479; doi:10.3389/fonc.2026.1883024)
Supplement: Supplementary Table 1 — Summary of plan complexity and 3D dose−distribution radiomic features.The table lists the feature categories, feature counts, and brief descriptions for all variables extracted in this study. Abbreviations correspond to the feature groups defined in the Methods. Abbreviations: GLCM, gray level co-occurrence matrix, GLDM, gray level dependence matrix, GLRLM, gray level run length matrix, GLSZM, gray level size zone matrix, NGTDM, neighboring gray tone difference matrix, FW, Field Width, PT, Projection Time, GP, Gantry Period, TT, Treatment Time, TL, Target Length, CS, Couch Speed, CT, Couch Translation, Nproj, Number of projections, Nrot, Number of rotations, MF, Modulation Factor, TTDF, Treatment time over fraction dose, mLOT, LOT mean, sdLOT, LOT standard deviation, mdLOT, LOT median, moLOT, LOT mode, minLOT, LOT minimum, maxLOT, LOT maximum, CLNSn, Cumulative LOT Number Score, CLNSpt,n, Cumulative LOT Number Score at Projection Time, kLOT, LOT kurtosis, sLOT, LOT skewness, mFLOT, sdFLOT, FLOT standard deviation, moFLOT, FLOT mode, mdFLOT, FLOT median, minFLOT, FLOT minimum, maxFLOT, FLOT maximum, CFNSn, Cumulative FLOT Number Score, LnNS, Leaves with n Open Nearest Neighbors, CLS, Closed Leaf Score, CLSin, Closed Leaf Score within the treatment area, nCC, Number of Connected Components, lengthCC, Length of the Connected Components, TA, Treatment Area, fDISC, Fraction of Discontinuous Projections, CLSin,area, the number of closed leaves within the treatment area, CLSin,disc, it is computed by considering the discontinuous projections is then divided by the number of discontinuous projections, CLSin,area,disc, the combination of the CLSin,area with the CLSin,disc, PSTV, Plan Sinogram Time Variation, LOTV, Leaf Open Time Variability, MI, Modulation Index, nOC, Number of Openings and Closures, EPSTV∆p,∆l, Extended Plan Sinogram Time Variation, mSI, the mean sinogram intensity, mdSI, the median sinogram intensity, sdSI, the standard deviation sinogram intensity, MSA, M [file DataSheet1.docx]

**Figure Legends**

**Figure S1.** Sensitivity analysis of the number of selected features used in recursive feature elimination (RFE).

**Table S1. Summary of plan complexity and 3D dose‑distribution radiomic features.**

|  | Feature | N | Description |
| --- | --- | --- | --- |
| Plan complexity features | TPS delivery | 13 | Pitch, FW, PT, GP, TT, TL, CS, CT, N_proj_, N_proj,period_, N_rot_, MF, TTDF |
|  | LOT statistics | 28 | Absolute LOT: mLOT, sdLOT, mdLOT, moLOT, minLOT,maxLOT, CLNS_100_, CLNS_50_, CLNS_30_,  CLNS_pt,20_,kLOT, sLOT, CLNS_20_  Relative LOT: mFLOT, sdFLOT, moFLOT, mdFLOT, minFLOT,maxFLOT,CFNS_5_, CFNS_10_,CFNS_25_,CFNS_50_,CFNS_75_, CFNS_90_,CFNS_95_,CFNS_98_,CFNS_100_ |
|  | Sinogram | 31 | Geometry:L0NS,L1NS,CLS,CLS_in_,L2NS, nCC,lengthCC,TA,fDISC,CLS_in,area_,LS_in,disc_, CLS_in,area,disc_, centroid  Modulation: PSTV, LOTV, MI,nOC, EPSTV1_1, EPSTV1_2, EPSTV1_10, EPSTV0_1, EPSTV1_0,ELOTV1, ELOTV2, ELOTV3, ELOTV4,ELOTV5, mSI, mdSI, sdSI, MSA |
| 3D dose-distribution radiomic features | shape | 14 | Voxel volume, Maximum 3D diameter, etc. |
|  | Statistical information | 162 | Skewness, Uniformity, etc. |
|  | GLCM | 216 | Joint average, joint entropy, etc. |
|  | GLDM | 126 | Gray level emphasis, Dependence entropy, etc. |
|  | GLRLM | 144 | Gray level variance, Run variance, etc. |
|  | GLSZM | 144 | Gray level variance, Zone variance, etc. |
|  | NGTDM | 45 | Complexity, Strength, etc. |

The table lists the feature categories, feature counts, and brief descriptions for all variables extracted in this study. Abbreviations correspond to the feature groups defined in the Methods.

Abbreviations: GLCM = gray level co-occurrence matrix, GLDM = gray level dependence matrix, GLRLM = gray level run length matrix, GLSZM = gray level size zone matrix, NGTDM = neighbouring gray tone difference matrix, FW=Field Width, PT=Projection Time, GP=Gantry Period, TT=Treatment Time, TL=Target Length, CS=Couch Speed, CT=Couch Translation, N_proj_=Number of projections, N_rot_=Number of rotations, MF=Modulation Factor, TTDF=Treatment time over fraction dose, mLOT=LOT mean, sdLOT=LOT standard deviation, mdLOT=LOT median, moLOT=LOT mode, minLOT=LOT minimum, maxLOT=LOT maximum, CLNS_n_=Cumulative LOT Number Score, CLNS_pt,n_=Cumulative LOT Number Score at Projection Time, kLOT=LOT kurtosis, sLOT=LOT skewness, mFLOT, sdFLOT=FLOT standard deviation, moFLOT=FLOT mode, mdFLOT=FLOT median, minFLOT=FLOT minimum, maxFLOT=FLOT maximum, CFNS_n_=Cumulative FLOT Number Score, LnNS=Leaves with n Open Nearest Neighbors, CLS=Closed Leaf Score, CLSin=Closed Leaf Score within the treatment area, nCC=Number of Connected Components, lengthCC=Length of the Connected Components, TA=Treatment Area, fDISC=Fraction of Discontinuous Projections, CLSin,area=the number of closed leaves within the treatment area, CLSin,disc=it is computed by considering the discontinuous projections is then divided by the number of discontinuous projections, CLSin,area,disc=the combination of the CLSin,area with the CLSin,disc, PSTV=Plan Sinogram Time Variation, LOTV=Leaf Open Time Variability, MI=Modulation Index, nOC=Number of Openings and Closures, EPSTV_∆p,∆l_=Extended Plan Sinogram Time Variation, mSI=the mean sinogram intensity, mdSI=the median sinogram intensity, sdSI=the standard deviation sinogram intensity, MSA=Mean Sinogram Asymmetry.

**Table S2.** Classification performance and test-set class distribution for Institution 1.

| **Criteria** | **Models** | **Test positive**  **(n)** | **Test negative**  **(n)** | **AUC (95% CI)** | **Sensitivity** | **Specificity** | **Accuracy** | **F1-score** |
| --- | --- | --- | --- | --- | --- | --- | --- | --- |
| γ 3%/2mm | PM | 51 | 6 | 0.469 (0.352-0.576) | 1.000 | 0.167 | 0.895 | 0.944 |
|  | DM | 51 | 6 | 0.604 (0.455-0.762) | 1.000 | 0.167 | 0.912 | 0.953 |
|  | HM | 51 | 6 | 0.774 (0.645-0.886) | 1.000 | 0.333 | 0.930 | 0.962 |
| γ 2%/2mm | PM | 48 | 9 | 0.668 (0.576-0.757) | 0.979 | 0.222 | 0.825 | 0.904 |
|  | DM | 48 | 9 | 0.622 (0.532-0.713) | 0.958 | 0.222 | 0.842 | 0.911 |
|  | HM | 48 | 9 | 0.820 (0.739-0.890) | 1.000 | 0.333 | 0.895 | 0.941 |

Results are shown for the 95% action limit under theγ3%/2 mm criterion and the 90% action limit under theγ2%/2 mm criterion. Positive and negative counts refer to the number of threshold-passing and threshold-failing plans in the independent test subset.

Abbreviations: AUC, area under the receiver operating characteristic curve; PM, plan model; DM, dose model; HM, hybrid model.

**Table S3.** Classification performance and test-set class distribution for Institution 2.

| **Criteria** | **Models** | **Test positive**  **(n)** | **Test negative**  **(n)** | **AUC (95% CI)** | **Sensitivity** | **Specificity** | **Accuracy** | **F1-score** |
| --- | --- | --- | --- | --- | --- | --- | --- | --- |
| γ 3%/2mm | PM | 40 | 2 | 0.737 (0.517-0.864) | 1.000 | 0.167 | 0.952 | 0.976 |
|  | DM | 40 | 2 | 0.838 (0.742-0.917) | 0.975 | 0.167 | 0.929 | 0.963 |
|  | HM | 40 | 2 | 0.938 (0.885-0.983) | 1.000 | 0.500 | 0.976 | 0.988 |
| γ 2%/2mm | PM | 39 | 3 | 0.574 (0.465-0.683) | 1.000 | 0.222 | 0.929 | 0.963 |
|  | DM | 39 | 3 | 0.568 (0.399-0.715) | 1.000 | 0.222 | 0.929 | 0.963 |
|  | HM | 39 | 3 | 0.825 (0.744-0.902) | 1.000 | 0.333 | 0.952 | 0.975 |

Results are shown for the 95% action limit under the γ 3%/2 mm criterion and the 90% action limit under the γ 2%/2 mm criterion. Positive and negative counts refer to the number of threshold-passing and threshold-failing plans in the independent test subset.

Abbreviations: AUC, area under the receiver operating characteristic curve; PM, plan model; DM, dose model; HM, hybrid model.

**Table S4.** Exact permutation test results for the hybrid model in Institution 2 under the γ 3%/2 mm criterion.

| Metric | Value |
| --- | --- |
| Number of test samples | 42 |
| Test positives (threshold-passing) | 40 |
| Test negatives (threshold-failing) | 2 |
| Observed AUC | 0.9375 |
| Number of permutations | 861 |
| Mean of permutation AUC distribution | 0.500 |
| 95th percentile of permutation distribution | 0.850 |
| 97.5th percentile of permutation distribution | 0.900 |
| Permutations with AUC ≥ observed AUC | 12 |
| One-sided p-value | 0.0139 |

The permutation test assessed whether the high AUC observed for the hybrid model could arise from random label assignments under the highly imbalanced class distribution. The number of threshold‑passing and threshold‑failing labels in the test set was held constant (40 and 2, respectively), and all C(42, 2) = 861 possible label configurations were enumerated. For each configuration, the AUC was recalculated while keeping the model‑predicted probabilities fixed. The one‑sided p‑value was defined as the proportion of permutations with AUC values greater than or equal to the observed AUC.

**Table S5.** Cross-institution validation results: AUCs for models trained on one institution and tested on the other.

| **Training institution → Testing institution** | **Gamma criterion** | **PM AUC** | **DM AUC** | **HM AUC** |
| --- | --- | --- | --- | --- |
| Institution 1 → Institution 2 | γ 3%/2 mm (95%) | 0.531 | 0.627 | 0.501 |
| Institution 1 → Institution 2 | 2%/2 mm, 90% | 0.555 | 0.463 | 0.717 |
| Institution 2 → Institution 1 | γ 3%/2 mm (95%) | 0.411 | 0.508 | 0.498 |
| Institution 2 → Institution 1 | γ 2%/2 mm (90%) | 0.549 | 0.560 | 0.544 |

For each direction, models were trained using the full dataset from the training institution only. The trained models were then directly applied to the complete dataset of the testing institution, which did not participate in model development. The γ 3%/2 mm and γ 2%/2 mm criteria correspond to action limits of 95% and 90%, respectively.
